# Early detection of cerebrospinal fluid/serum glucose ratio: A promising value for mortality prognosis in patients with acute bacterial meningitis

**DOI:** 10.1002/jgf2.70004

**Published:** 2025-02-20

**Authors:** Nasrin Khodashenas, Rouzbeh Rajaei Ghafouri, Asghar Jafari Rouhi, Moloud Balafar

**Affiliations:** ^1^ Emergency and Trauma Care Research Center Tabriz University of Medical Sciences Tabriz Iran

**Keywords:** cerebrospinal fluid, glucose, meningitis bacterial, mortality, spinal puncture

## Abstract

**Background/Aims:**

Given that acute bacterial meningitis (ABM), as community‐acquired meningitis, is considered a life‐threatening medical emergency, early diagnosis and immediate onset of antibiotic therapy are of great importance. Here, we aimed to compare both the laboratory and glucometer assessment of cerebrospinal fluid (CSF)/serum glucose ratio in patients with suspected ABM. In addition, the prognostic value of the CSF/serum glucose ratio on the mortality rate was also evaluated.

**Methods:**

In this cross‐sectional, single‐center study, all demographic and clinical characteristics of patients who attended the emergency department were evaluated. A total of 200 patients with clinical suspicion of ABM underwent lumbar puncture (LP) and were simultaneously recruited for CSF and serum glucose measurement using both laboratory tests and a point‐of‐care glucometer device. Data analysis was performed using SPSS *ver*. 20.0.

**Results:**

Out of 200 suspected subjects with a mean age of 52.66 ± 6.64 years (male = 113, 56.5%), 23 patients (11.5%) were identified with confirmed ABM. The study findings showed a close correlation between the laboratory and glucometer‐measured CSF/serum glucose ratio. The sensitivity and specificity of the glucose ratio measured by the laboratory test were 91.3% and 86.4%, respectively. A similar result was also observed by glucometer measurement (Sensitivity: 91.3%, Specificity: 89.3%, *p* < 0.001). However, the mean time needed for CSF glucose measurement was significantly longer in the laboratory method compared to the glucometer‐based assessment (*p* < 0.0001). Notably, the CSF/serum glucose ratio was considerably lower in deceased patients (*n* = 9, *p* < 0.05).

**Conclusion:**

Despite the similar accuracy of the CSF/serum glucose ratio using either a laboratory test or glucometer for ABM diagnosis, the present study highlighted that the laboratory turnaround time was significantly improved using a glucometer, which could be considered a reliable aid to the prognosis of mortality.

## Graphical abstract

This study revealed that a glucometer‐based measurement significantly improved the turnaround time required for CSF/serum glucose ratio measurement compared to conventional laboratory tests, which could be considered a reliable aid to the mortality prognosis.

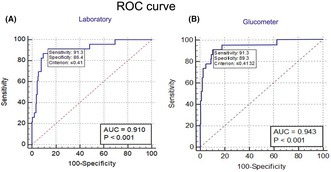

## 1 INTRODUCTION

Acute community‐acquired bacterial meningitis (ABM) is defined as a life‐threatening condition requiring immediate diagnosis (up to 60 min after admission),^1^, ^2^, ^3^ which is caused by various bacterial pathogens consisting of *Streptococcus pneumoniae, Neisseria meningitidis, Staphylococcus epidermidis, Staphylococcus aureus, and Haemophilus influenzae type b (Hib)*.^4^, ^5^, ^6^, ^7^ The incidence of stroke, hearing loss, and permanent brain damage is known as the most serious complications observed following the ABM.^8^, ^9^, ^10^ Cerebrospinal fluid (CSF) cell count and the measurement of both protein and glucose concentrations in the CSF are performed for ABM diagnosis.^11^ In addition, blood cultures or other non‐cultural diagnostic methods might help detect causative pathogens.

Besides the typical clinical manifestations, CSF cytochemical characteristics, one of the most important modalities for the diagnosis of meningitis, are performed using a Gram stain‐specific probe‐based real‐time PCR (positive result >1 × 10^3^ cells/ ml), CSF culture (with 71% sensitivity), and antigen assays e.g., latex agglutination test (86% sensitivity).^12^ For detecting the causative microbial pathogens, the CSF pressure record should also be employed, and simultaneously, an immediate empirical antibiotic therapy should be initiated in suspected individuals.^13^, ^14^

It has been well‐established that the progression of bacterial meningitis is directly associated with acute hyperglycemia due to the elevated levels of circulating cytokines.^15^ In this regard, available evidence also indicates that high glucose levels are associated with poor outcomes in neuro‐infectious diseases such as meningitis.^16^

Noteworthy, a linear correlation has also been found between serum and CSF glucose in healthy people, in which the normal levels of glucose in CSF are estimated at approximately 60% of the serum glucose.^17^ In this respect, the CSF/serum glucose ratio could be changed following some pathological conditions, such as ABM, aseptic meningitis, diabetes, and ventricular shunt.^17^

Using cost–benefit and accessible modalities, especially in healthcare centers with limited facilities, is of major importance for early diagnosis and treatment of ABM. Hence, the main aim of this study was to compare the application of a glucometer, a fast‐tracked modality, with the conventional laboratory test to measure the CSF/serum glucose ratio as a potential indicator of ABM, which is associated with better quality of care and optimum management, especially in high‐volume emergency departments (EDs). In this regard, the results of this study can also be applied in resource‐limited centers to minimize requests for laboratory measurements and lessen the need for empiric antimicrobial therapy. In the current study, we first explored the association between serum and CSF levels of glucose in ABM‐suspected patients measured by two different modalities. In addition, the CSF/serum glucose ratio and related specificity and sensitivity were compared in the two modalities. We also determined whether the CSF/serum glucose ratio is associated with the mortality rate in diagnosed patients.

## 2 METHODS

### 2.1 Study population

In this cross‐sectional, single‐center study, all patients who underwent lumbar puncture (LP) procedures for ABM diagnosis, from December 2020 to August 2021, were included. Based on a previous study performed by Rousseau et al., the sample size was calculated considering *α* = 0.05, *p* = %95, and *d* = 0.06,^18^ and the final 200 patients were included in a 1000‐bed tertiary referral hospital with more than 120,000 admitted patients per year in the ED. The clinical and demographic characteristics of the 200 patients, including age, gender, CSF, and serum glucose levels measured by two modalities, were recorded at the time of admission The confirmed diagnosis of ABM was defined as bacterial growth in the CSF culture or pathogen recognition by Gram staining in the CSF specimen. According to the study aims, the possible differences between the mean CSF/serum glucose ratio and the mean time required for measurement were assessed in both modalities.

## 3 ETHICS APPROVAL AND CONSENT TO PARTICIPATE

This study was approved by the ethics committee of Tabriz University of Medical Sciences [Ethics Committee Review (2021) No. IR.TBZMED.REC. 1399.768]. Following the explanation of all aspects of the study, written informed consent was obtained from all participants for data analysis, either from conscious patients or relatives of unconscious and un‐oriented individuals. This study was performed according to the ethical standards as laid down in the 1964 Declaration of Helsinki and its later amendments or comparable ethical standards. Of note, the information of all patients was confidential, and no additional cost was incurred to them.

### 3.1 Inclusion/exclusion criteria

All patients referred to the ED, with ages between 18 and 65 years old, who were suspected to have ABM with clinical symptoms, including fever, headache, and decreased level of consciousness, were regarded as inclusion criteria. While the patients aged >65 and <18 years, a history of LP for other purposes, and antibiotic therapy for 2 weeks before LP were not eligible to be included. In addition, aseptic meningitis was defined for patients with pleocytosis >5 cells/μL, no pathogen grew in CSF culture, and negative results of Gram staining.^19^

### 3.2 Data processing and statistical analysis

Mean ± *SD* and frequency (%) were used for continuous variables. Relative frequencies were presented for each variable. The normal distribution was estimated using the Kolmogorov–Smirnov test. The two‐paired student *t‐test* was also applied to compare the mean of quantitative variables with normal distribution. Sensitivity, specificity, and positive and negative predictive values were calculated using the MedCalc statistical software *version 22.012*. Finally, the data was analyzed by the SPSS software *ver. 23* packages (SPSS Inc). The *p*‐value level <0.05 was considered statistically significant.

## 4 RESULTS

In the current study, 200 participants with a mean age of 52.66 ± 6.64 years old (min = 18, max = 65) who were suspected of ABM were included. Also, the prevalence of male gender was higher (*n* = 113, 56.5%). In Table 1, demographic characteristics and the causative pathogens of ABM were listed. The glucose levels of serum and CSF measured by both laboratory test and glucometer modalities in the highly suspected patients have been shown in Table 2. The results of this study indicated no significant differences between the two methods. In addition, the mean time of glucose measurement was compared between the two methods. The data showed that the turnaround time needed for glucose measurement was shorter following the glucometer method (Table 2).

**TABLE 1:** Demographic data of patients with suspected ABM and the causative microorganisms involved in disease pathogenesis (*n* = 200).

Age	Mean age = 52.66 ± 6.64 (min = 18, max = 65 years old)
Gender	87 females, 43.5%
Clinical manifestations	Fever, headache with any degree of decreased level of consciousness

Microorganisms	Patients (*n* = 23)
*Streptococcus pneumoniae*	11 (47.8%)
*Haemophilus influenzae*	5 (21.7%)
*Other gram‐negative bacilli*	4 (17.3%)
*N. meningitides*	1 (4.3%)
*Staphylococcus aureus*	1 (4.3%)
*Listeria monocytogenes*	1 (4.3%)

**TABLE 2:** The quantitative results of glucose measurement in patients with suspected ABM (*n* = 200).

BG (mg/dL)	Mean ± *SD*	Min‐max	*p* value
Lab	109.66 ± 20.91	67.00–163.00	0.05>
Glu	101.59 ± 18.18	66.00–160.00	
CSF glucose (mg/dL)	Mean ± *SD*	Min‐Max	0.05>
Lab	62.16 ± 21.08	7.00–123.00	
Glu	54.34 ± 19.20	9.00–124.00	
Turn‐around time (min)	Mean ± *SD*	Min‐Max	0.05<
Lab	229.40 ± 69.80	29.00–409.00	
Glu	7.14 ± 3.04	0.40–14.50	

Abbreviations: BG, Blood Glucose; CSF, Cerebrospinal fluid; Glu, Glucometer; Lab, Laboratory; Max, Maximum; Min, Minimum; *SD*, Standard Deviation.

The assessment of the CSF/serum glucose ratio is one of the reliable indicators to identify ABM in suspected individuals.^20^ Based on our findings, the ratio calculated in both laboratory and glucometer methods was 0.58 ± 0.22 and 0.53 ± 0.15, respectively (Table 3), in which a significant difference was not observed (*p* > 0.05). To ascertain the mean time for CSF and serum glucose measurement, the independent *t‐test* was also applied. Noticeably, the results noted that the mean turn‐around time using a glucometer was significantly shorter in comparison to the laboratory test (7.14 ± 3.04 [95% confidence interval, CI, 6.71–7.56] vs. 229.40 ± 69.80 [95% CI 219.67–239.13]; *p* < 0.0001; Table 3).

**TABLE 3:** Comparison of CSF/serum glucose ratio and mean turnaround time measured by laboratory and glucometer (*n* = 200).

Ratio	Mean ± *SD*	Min‐max	*p*‐value
Lab	0.58 ± 0.22	0.07–0.97	*p* > 0.05
Glu	0.53 ± 0.15	0.09–0.91	
Turn‐around time (min)	Arithmetic Mean ± *SD*	95% CI	*p* < 0.0001^a^
Lab	229.40 ± 69.80	219.67–239.13	
Glu	7.14 ± 3.04	6.71–7.56	

Abbreviations: Glu, Glucometer; Lab, Laboratory; Max, Maximum; Min, Minimum; min, minute; *SD*, Standard Deviation.

^a^ Analyzed by independent *T*‐test, Two‐tailed probability.

**FIGURE 1:**
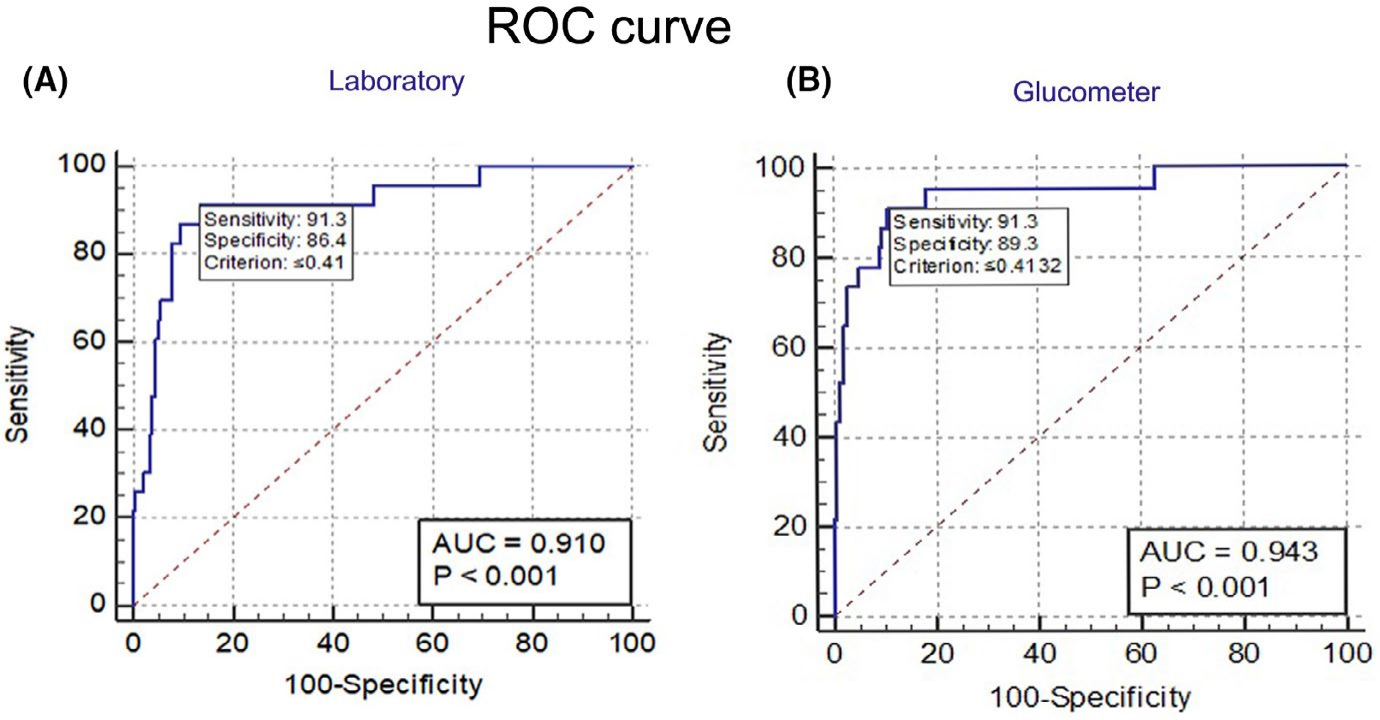
The role of CSF/serum glucose ratio in ABM diagnosis. (A) the laboratory‐related ROC curve. (B) the glucometer‐related ROC curve.

According to the results of diagnostic tests, out of 200 patients, 23 (11.5%) were confirmed with ABM. The sensitivity, specificity, area under the curve (AUC), and cut‐off values for the CSF/serum glucose ratio have also been shown in Figure 1A. Considering the disease prevalence (11.5%), the mean of AUC, sensitivity, and specificity shown in the ROC curve in the laboratory test was 0.91, 91.30, and 86.44, respectively [*p* < 0.001 with a 95% CI 0.90–0.97, Figure 1A]. Similarly, the AUC, sensitivity, and specificity, represented by the ROC curve, were 0.94, 91.30, and 89.27, respectively, following glucometer measurement (*p* < 0.001 with 95% CI 0.86–0.94, Figure 1B). In this study, the cut‐off point value for the CSF/blood glucose ratio as an indicator of the ABM ratio was 0.41 for both laboratory and glucometer measurements.

**FIGURE 2:**
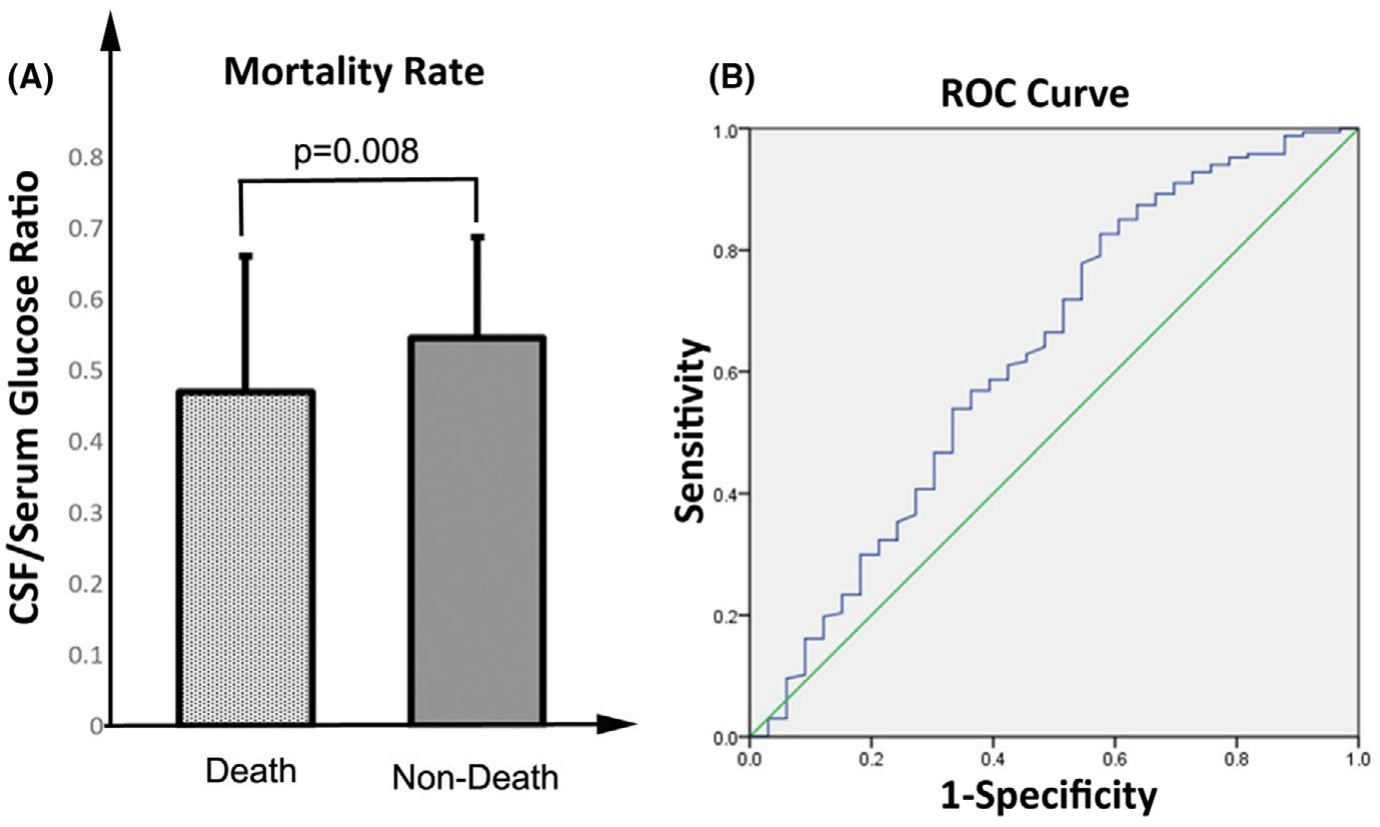
The association between CSF/serum glucose ratio and mortality. (A) the comparison of CSF/serum glucose ratio between dead and non‐dead groups (Dead vs. non‐dead *p* value <0.001), (B) A ROC presented an appreciable sensitivity and specificity regarding the potential of CSF/serum glucose ratio as a prognostic value.

Mortality is one of the concerning issues among the patients admitted with ABM. The results of the current study also reported nine deaths. In this regard, the association between the mortality rate and CSF/serum glucose ratio was also evaluated. Interestingly, the results showed that the CSF/serum glucose ratio in the non‐death group was higher than in dead patients (0.546 ± 0.141 vs. 0.470 ± 0.191; *p* = 0.008; Figure 2A). In addition, the results of the ROC curve with AUC = 0.629 also revealed that the CSF/serum glucose ratio has the potential to be a prognostic value with acceptable sensitivity and specificity (Figure 2B).

## 5 DISCUSSION

The CSF/serum glucose ratio appears to be a diagnostic value to determine ABM in suspected patients admitted to the ED. Given the importance of timely identification of ABM for therapeutic benefits in the clinical setting,^21^ we aimed to compare the accuracy and velocity of the diagnostic modalities to measure glucose in serum and the CSF of suspected patients who underwent LP. Our findings uncovered no significant differences between the measured glucose levels (*p* > 0.05). The time required for a glucometer‐based measurement was remarkably shorter than that of the laboratory test. In addition, we found that the CSF/serum glucose ratio could be considered a prognostic value in the mortality rate of ABM patients.

Schut et al. reported that the majority of patients with ABM were hyperglycemic (fasting blood glucose: 7.8 mmol/L or 140 mg/dL) on admission and were at higher risk of unfavorable outcomes.^22^ In this subgroup, the higher serum levels of glucose are most likely to occur due to a physical stress response and the impairment of the blood‐glucose regulatory system.^22^ In line with our findings, Tamune et al. also demonstrated that the CSF/serum glucose ratio could be considered a diagnostic and accurate indicator for ABM detection.^20^ Mortality is considered one of the adverse events following delayed antibiotic therapy in patients with ABM.^23^ In this sense, optimal management of patients, including timely diagnosis to measure CSF and serum glucose and early antibiotic therapy (door‐to‐antibiotic time <6 h) could significantly decline the mortality rate. Martín‐Cerezuela et al. also indicated three factors that were independently associated with ABM‐induced mortality, consisting of delay in antibiotic therapy initiation, sepsis‐related organ failure assessment (SOFA) score, and immediate treatment failure.^24^ To prove a better turnaround time obtained by glucometer measurement, we also highlighted that the CSF/serum glucose ratio may be a strong predictor of mortality, which mostly occurred upon septic shock in subgroups with positive BM. Intriguingly, we found a negative association between the CSF/serum glucose ratio and mortality rate, in which the levels of the CSF/serum glucose ratio were significantly lower in the deceased group.

A systematic review performed in 2016 demonstrated that CSF lactate, CSF glucose, the proportion of polymorphonuclear leukocytes, and serum pro‐calcitonin (PCT) could also be considered independent factors to predict meningitis with bacterial etiology.^25^ Also, in a recent retrospective study, it was revealed that the Glasgow coma score (GCS) between 1 and 4 was applied to determine undesirable outcomes in association with ABM following a routine diagnostic and therapeutic schedule.^26^ In this study, beyond the blood culture, the patients who were positive through laboratory and imaging examinations were also involved. The results showed that the rate of unfavorable outcomes (mainly headaches) was higher than BM‐induced mortality (40.5% vs. 8.8%).^26^ The accuracy of glucometer‐detected hypoglycorrhachia, defined as a CSF glucose level <45 mg/dL or a CSF/serum glucose ratio ≤0.5, was previously investigated in pediatrics and neonates with meningitis.^27^ Given that the time of glucose measurement is a critical issue, the delays in CSF analysis can affect the glucose value.

For CSF glucose assessment, Rousseau et al. also declared that ABM diagnosis was accurately achieved using a point‐of‐care glucometer at the bedside.^18^ Similar to our findings, they also reported a significant difference in the case of median turnaround time between the glucometer and central laboratory (*p* < 0.0001).^18^ Furthermore, the cut‐off value reported for the CSF/serum glucose ratio for the bedside glucometer was 0.46, with a sensitivity and specificity of 94.1% and 91%, respectively, which is in line with our findings.^18^

In another study, Alkhalifah et al. also noted appreciated sensitivity and specificity of glucometer‐measured CSF and serum glucose in suspected pediatrics and neonates with meningitis.^27^ Therefore, the glucometer can be suggested as an accessible and accurate tool with rapid measurement of CSF and serum glucose even with slight overestimation. In parallel with our results, Sanjith et al. also found a statistically remarkable correlation between ABM and CSF glucose using both conventional laboratory assessment and glucometer (OR, 0.976; 95% CI 0.957–0.993; *p* = 0.0165) vs. (OR, 0.975; 95% CI 0.956–0.996; *p* = 0.0066).^28^

## 6 STUDY LIMITATION

As the study limitations, the sample size was rather small. In addition, given the recent advantage of glucometer application, as the point‐of‐care tool, large‐scale research is required to further confirm the cost–benefit aspects of glucometer‐based measurement. As a future perspective, the accuracy of laboratory tests and glucometer‐measured serum and CSF glucose levels could be compared in other conditions that are related to glucose metabolism, as well as the assessment of CSF/serum glucose ratio in lumbar and ventricular compartments to develop the application of glucometer as an alternative aid, especially in limited‐resource medical centers.

## 7 CONCLUSION

Despite the accurate estimation of the CSF/serum glucose ratio using both laboratory tests and a glucometer at the bedside, the study findings indicated that the turnaround time required for glucose measurement was significantly improved by a cheap point‐of‐care method (glucometer‐based measurement). As a clinical implication, using cost–benefit and user‐friendly tools such as a glucometer with appreciated accuracy would be a practical aid for physicians and other healthcare providers in the early diagnosis of ABM, especially in centers with limited resources and facilities. Beyond the cost–benefit effect of glucometer‐based assessment, timely diagnosis of ABM and treatment with antibiotics and corticosteroids are associated with improved clinical outcomes. Furthermore, the CSF/serum glucose ratio could be considered a reliable indicator regarding the mortality prognosis.

## AUTHOR CONTRIBUTIONS

RRGh, designed and conceptualized the study, NKh contributed to data collection, RRGh contributed to data interpretation; MB, performed the process; AJR, analyzed the data and screened the articles; NKh, drafted the manuscript. All authors: contributed to the manuscript, and read and approved the final manuscript.

## FUNDING INFORMATION

None.

## CONFLICT OF INTEREST STATEMENT

None declared.

## ETHICS APPROVAL STATEMENT

The protocol of this study was approved by the medical ethics committee of our institution.

## PATIENT CONSENT STATEMENT

Informed consent was obtained from the participants.

## Data Availability

All Data and material collected during this study are available from the corresponding author upon reasonable request.
